# The Core Components and Instruments of the Therapeutic Relationship in Musculoskeletal Physiotherapy: A Systematic Integrative Review

**DOI:** 10.1002/msc.70219

**Published:** 2026-03-30

**Authors:** Sini Puustinen, Tarja Kvist, Minna Stolt

**Affiliations:** ^1^ Department of Nursing Science University of Eastern Finland Kuopio Finland; ^2^ Karelia University of Applied Sciences Joensuu Finland; ^3^ Wellbeing Services County of Central Finland Jyväskylä Finland; ^4^ Department of Nursing Science University of Turku Turku Finland; ^5^ Wellbeing Services County of Satakunta Pori Finland

**Keywords:** instruments, musculoskeletal, physiotherapy, systematic review, therapeutic relationship

## Abstract

**Background:**

The therapeutic relationship is meaningful and valuable to patients in rehabilitation and is positively associated with improved rehabilitation outcomes. Despite its central role, a comprehensive understanding of what constitutes the therapeutic relationship in physiotherapy and how it can be assessed is yet to be achieved.

**Objective:**

This study aimed to identify the core components of the therapeutic relationship and the patient‐reported instruments used to assess it in musculoskeletal physiotherapy.

**Methods:**

A systematic integrative review was conducted. Six databases (CINAHL, PubMed/Medline, Scopus, Web of Science, The Cochrane Library, and PEDro) were searched. Two reviewers independently conducted study selection and methodological quality appraisal. Data were synthesised using inductive qualitative content analysis.

**Results:**

Eighteen studies were included. Two core components and six subcomponents of the therapeutic relationship in musculoskeletal physiotherapy were identified: (1) therapeutic partnership (partnership and therapeutic communication) and (2) collaborative person‐centred physiotherapy (collaboration; a holistic and individualised approach; coherent, competent, and credible physiotherapy; and empowerment support). Six instruments used to assess therapeutic relationships in musculoskeletal physiotherapy were identified.

**Conclusions:**

In musculoskeletal physiotherapy, the therapeutic relationship is a multidimensional and dynamic construct encompassing therapeutic partnership and collaborative person‐centred practice. Physiotherapists can actively influence the therapeutic relationship by utilising multimodal communication, implementing person‐centred care strategies, and supporting patients' agency and empowerment. Existing patient‐reported therapeutic relationship instruments capture these components only partially. Future research is needed to strengthen conceptual clarity and refine both the operationalisation and assessment of the therapeutic relationship in musculoskeletal physiotherapy.

## 1 Introduction

The therapeutic relationship (TR) plays a fundamental role in the provision of evidence‐based, person‐centred healthcare and rehabilitation (Hamovitch et al. 2018; Moreno‐Poyato et al. 2021). The TR refers to the relational processes involved in rehabilitation, which may act independently or in combination with specific interventions (Babatunde et al. 2017). Several studies highlight the meaningfulness and value of the TR for patients as part of the rehabilitation process (Liddiard et al. 2023; Morera‐Balaguer et al. 2021) and indicate a positive association between the TR and improved rehabilitation outcomes (Hall et al. 2010; Kinney et al. 2020; Sherriff et al. 2022). Despite the central role of the TR in healthcare and rehabilitation, its core components and assessment methods remain underexplored in physiotherapy, highlighting the need for further exploration to advance meaningful, person‐centred care and optimise therapeutic outcomes.

In the literature, TR and related concepts, such as therapeutic alliances (TAs) and working alliances (WAs) are often used interchangeably. A TA refers to the affective and collaborative bond between patient and therapist, while a WA emphasises agreement on goals and tasks within the therapeutic process (Bordin 1979; Gelso 2014). However, the TR is considered a broader concept, with alliances positioned within its framework (Gelso 2014; Horvath 2018). These concepts have been extensively studied in psychotherapy (Bordin 1979; Gelso 2014; Horvath 2018). Consequently, physiotherapy research has largely borrowed psychotherapy's conceptualisation, frameworks, and instruments for assessing the TR, adapting them where necessary. However, these adaptations may not fully capture elements central and specific to physiotherapy, such as the uniqueness of the process and the connection through the body, including exercise, manual therapy, and therapeutic touch. Therefore, previous studies have identified the need for a physiotherapy‐specific exploration of the TR construct (Babatunde et al. 2017; McCabe, Miciak, Roduta Roberts, Sun, et al. 2022).

In physiotherapy, growing interest in the TRs reflects a broader shift towards holistic and person‐centred care (Daluiso‐King and Hebron 2022; Wijma et al. 2017), especially in the management of musculoskeletal (MSK) conditions (Ernstzen et al. 2022). MSK conditions affect approximately 1.7 billion people globally, encompassing over 150 different conditions characterised by pain and functional impairment, representing a significant burden to individuals and healthcare systems (El‐Tallawy et al. 2021; WHO 2022). Although the TR has been explored in rehabilitation contexts (Bishop et al. 2021; Heredia‐Callejón et al. 2023), occupational therapy, and physiotherapy (Babatunde et al. 2017; Søndenå et al. 2020), few reviews have examined the factors specific to MSK physiotherapy that influence the TR (Kinney et al. 2020; O’Keeffe et al. 2016) or the instruments used to assess it in this context (Babatunde et al. 2017; Gutiérrez‐Sánchez et al. 2021). These reviews have focused on factors influencing patient‐therapist interactions in MSK physiotherapy (O’Keeffe et al. 2016) and TAs in physiotherapy for chronic MSK conditions (Kinney et al. 2020). The factors identified as most influential for the TR included the physiotherapist's interpersonal, communication, and practical skills, the provision of individualised patient‐centred care, and organisational and environmental factors (Kinney et al. 2020; O’Keeffe et al. 2016). However, a recent article has highlighted that the physiotherapy literature uses inconsistent terminology to define and construct the TR and determine the practices that contribute to it (Cole et al. 2025).

A comprehensive understanding of the TR's components and the instruments for its assessment is essential for developing physiotherapy towards a more holistic and person‐centred approach. Such an understanding would also enable physiotherapists to integrate elements of the TR into multidimensional MSK physiotherapy. Therefore, this study explored the existing literature to identify the core components of the TR and the patient‐reported instruments used to assess it in MSK physiotherapy.

## 2 Methods

We employed a systematic integrative review strategy, synthesising evidence from quantitative, qualitative, and mixed‐method studies to provide a comprehensive understanding of the TR in MSK physiotherapy from a broad perspective (Hopia et al. 2016; Oermann and Knafl 2021). The five‐stage integrative review method described by Whittemore and Knafl (2005) was adopted, comprising problem identification, literature search, methodological quality appraisal, data analysis, and the presentation and synthesis of findings. Reporting followed the PRISMA guidelines (Page et al. 2021). The review was registered in PROSPERO on September 23, 2024 (CRD42024588504).

### 2.1 Search Strategy and Study Selection

A systematic literature search was conducted in six electronic databases (CINAHL, PubMed/Medline, Scopus, Web of Science, The Cochrane Library, and PEDro) using Medical Subject Headings (MeSH) and other TR‐related terminology. The screening and selection of relevant studies were conducted in two phases using Covidence systematic review software (Veritas Health Innovation, Melbourne, Australia; available at www.covidence.org, 2024) guided by the predefined inclusion and exclusion criteria (Table 1). First, two reviewers (S.P. and M.S.) conducted an independent screening of titles and abstracts. Second, two reviewers (S.P. and M.S. or T.K.) independently reviewed the full‐text articles. The reasons for exclusion were recorded in Covidence. Disagreements between the reviewers were resolved through discussion, after which they reached a final consensus. Additionally, the reference lists of the selected studies were manually screened to identify all relevant studies on the topic.

**TABLE 1:** Inclusion and exclusion criteria for abstract screening and full‐text review.

Inclusion	Exclusion
Adults (age ≥ 18 years) with musculoskeletal related disorders or pain or	Children, adolescents or paediatric patients, experts, teachers, students, nurses or other healthcare professionals.
Physiotherapists working with adults with musculoskeletal related disorders or pain.	Patients or clients with neurological or cardio‐respiratory conditions, stroke, amputation, primary mental health disorders, HIV or cancer.
A description of — the components of the therapeutic relationship in musculoskeletal physiotherapy and/or — the patient‐reported measures of the therapeutic relationship that have been used or tested in musculoskeletal physiotherapy.	
Physiotherapy in in‐person physiotherapy setting (e.g. hospital, primary healthcare, public or private, and community context).	Remote physiotherapy setting or psychotherapy settings.
Primary, empirical, peer‐reviewed qualitative, quantitative and mixed methods studies in health and social sciences research field with the scope of therapeutic relationship which provide a description of Components of therapeutic relationship in musculoskeletal physiotherapy and/or Patient‐reported measures of the therapeutic relationship that have been used or tested in musculoskeletal physiotherapy. The concept of therapeutic relationship, or its synonyms/related concepts such as therapeutic alliance, working alliance, therapeutic bond, professional‐patient relationship, professional‐client relationship, patient‐therapist alliance, patient‐therapist relationship have to be considered in the title, aim/purpose or results as well as in the theoretical background. Publication period: 2014–2024. Language: English, Swedish, or Finnish.	Grey literature, conference abstracts, books chapters, editorials, dissertations and commentaries and
	Reviews (e.g. systematic reviews, scoping reviews, mixed‐method reviews, narrative reviews, and meta‐analyses and syntheses).

### 2.2 Quality Appraisal

The quality of the included studies was appraised using the Mixed Methods Appraisal Tool (MMAT, version 2018), which is designed for reviews employing diverse methodologies (Hong, Fàbregues, et al. 2018). The MMAT includes two screening questions and five methodology‐specific questions, with the rating options ‘yes’, ‘no’, and ‘can't tell’. The MMAT does not provide a summative numerical score. Instead, individual ratings for each criterion are recommended to appraise methodological strengths and weaknesses (Hong, Faberques, et al. 2018). Two reviewers (S.P. and M.S. or T.K.) independently conducted quality appraisal, reaching consensus through discussion.

### 2.3 Data Synthesis and Analysis

Inductive qualitative content analysis was adopted to synthesise and analyse the findings from the included studies (Graneheim et al. 2017; Lindgren et al. 2020). We selected this approach because the data were primarily descriptive, making qualitative content analysis suitable for systematically summarising and categorising content rather than interpreting underlying meanings, which is more typical of thematic analysis (Lindgren et al. 2020).

First, all articles were read repeatedly to ensure familiarity with the data. During this process, we selected text segments (such as words or sentences) from the articles' results sections describing the components of the TR in MSK physiotherapy and the instruments used to assess it, treating them as meaning units. For example, a result such as ‘learning strategies for self‐management in everyday activity’ was considered a meaning unit. Next, these meaning units were extracted and coded, yielding 54 codes for TR components and six for the instruments. Following this, codes related to TR components were compared for similarities and differences and grouped into six subcategories. These subcategories were further abstracted into two categories. Subcategories and categories were named to describe their content, ensuring alignment with the underlying data. An example of the data analysis process is presented in Table 2. The first reviewer (S.P.) performed the data extraction, coding and abstraction; the review group (S.P., M.S., T.K.) then verified the results for accuracy and completeness. Ultimately, this process identified two core components of the TR in MSK physiotherapy, comprising two and four subcomponents, and six instruments for its assessment.

**TABLE 2:** An example of the qualitative content analysis process.

Meaning unit	Code	Subcategory	Category
Therapeutic partnership			
Bond	Bond	Partnership	
Getting to know each other/making connections	Connection		
Gaining trust for and confidence in the physiotherapist and being seen as an individual were initially deemed necessary for creating this alliance	Trust		
	Confidence		
	Being seen as an individual		
Active listening	Verbal communication	Therapeutic communication	
Therapists, regardless of their treatment orientations, also recognise that touch is a very important aspect of connecting in the therapeutic relationship with the power to “… make or break…” (PT‐A) a relationship	Body communication		
Collaborative person‐centred physiotherapy			
Sharing of information and power	Shared decision‐ making	Collaboration	
Agreement on the goals of the treatment	Agreement on goals	Coherent, competent, and credible physiotherapy	
Professional aspects: Skill, competence, technical experience and knowledge	Competence of the physiotherapist		
Treatment credibility	Credible care		
Seeking to understand human and how complaint/condition affects life holistically	Holistic understanding	Holistic and individualised approach	
Participants described how the exercises, activities and other treatment modalities in the treatment process were explained, practiced and checked so that the participants felt the programs suited them	Individualised care		
Learning strategies for self‐management in everyday activity	Learning self‐management strategies	Empowerment support	
Several participants elaborated on the significance of the therapist's reassurance in their confidence to relax, stop worrying, and resume everyday function and physical activity	Physiotherapist's reassurance		

## 3 Results

**FIGURE 1:**
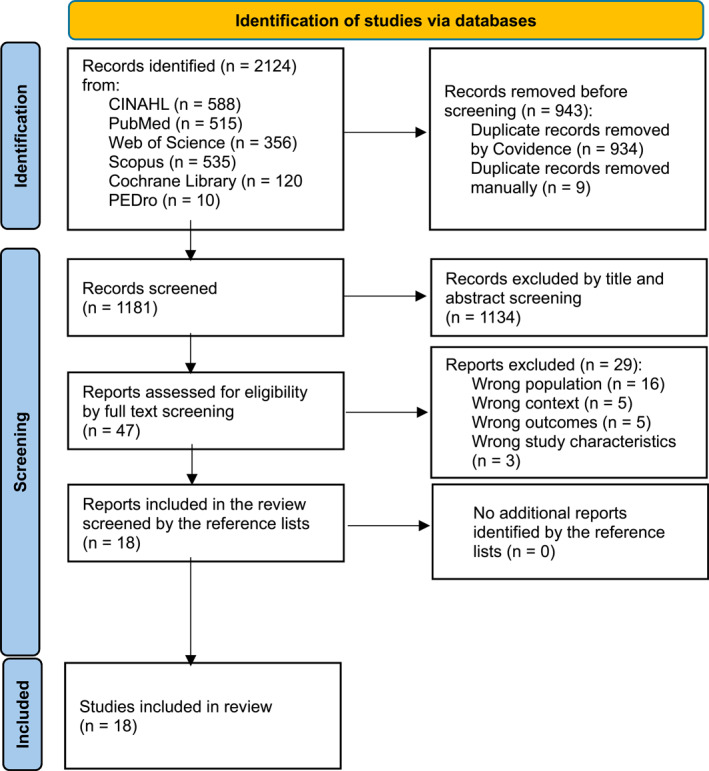
The PRISMA flow diagram, 2020 statement: An updated guideline for reporting systematic reviews (Page et al. 2021). This work is licenced under CC BY 4.0. To view a copy of this licence, visit https://creativecommons.org/licenses/by/4.0/.

A total of 2124 records were identified from six databases. After we removed duplicates and screened titles and abstracts, 47 articles were considered for eligibility in the full‐text review. Ultimately, 18 studies were included in the review (Figure 1). The included studies covered a variety of international contexts and methodological approaches and were published between 2014 and 2024. The designs included qualitative (e.g., hermeneutic, interpretive description, and exploratory), quantitative (cohort, cross‐sectional, and experimental), and mixed‐methods studies, and one methodological instrument development study. Participants included patients with diverse acute or chronic MSK disorders (*n* = 1861) and physiotherapists treating these conditions (*n* = 49). Table 3 presents the characteristics of the included studies and the data relevant to the review questions.

**TABLE 3:** Study characteristics of the included studies and the data relevant to the review questions.

Authors and publication year	Aim	Methods and design	Participants (*n*)	The core components of the therapeutic relationship in musculoskeletal physiotherapy	Patient‐reported instruments used to assess the therapeutic relationship in musculo‐skeletal physiotherapy	Quality appraisal^a^(MMAT, ●‐●●●●●)
Alodaibi et al. (2021)	To examine the relationship between TA assessed during a physiotherapy (PT) episode of care for patients with low back pain (LBP) and functional outcome at the conclusion of care.	Quantitative observational study.	Patients with acute or chronic LBP participating in PT (*n* = 676).	Agreement on the goals of the treatment,	Working alliance inventory‐short revised: WAI‐SR.	●●●
				Agreement on the tasks, the personal bond between patient and provider		
Beneciuk et al. (2021)	To identify patient‐ and physiotherapist ‐level predictors for TA at the end of an episode of PT for knee or LBP.	Quantitative observational study.	Patients (*n* = 447) with acute or chronic episodes of non‐operative or postoperative care for knee pain (*n* = 189) or LBP (*n* = 252).	Sharing of information and power	Working alliance inventory‐short revised: WAI‐SR.	●●●●
Calner et al. (2021)	To explore and describe the PT treatment experiences of persons with persistent MSK pain.	Qualitative exploratory study.	Patients with persistent MSK pain in neck, back, or shoulders (*n* = 11).	Feeling safe		●●●●●
				Being seen as an individual		
				Good dialogue with the physiotherapist		
Cosgrove and Hebron (2021)	To explore MSK physiotherapists conceptions towards the management of LBP.	Qualitative phenomenographic inspired approach.	Physiotherapists treating LBP (*n* = 6).	Communication		●●●
				Trust		
Fuentes et al. (2014)	To compare the effect of enhanced versus limited TA on pain intensity and muscle pain sensitivity in patients with chronic LBP receiving either active or sham interferential current therapy (IFC).	Quantitative double‐blind, placebo‐controlled experimental study.	Patients with chronic LBP (*n* = 117).	Verbal behaviours	Subscale of working alliance of the pain rehabilitation expectations scale: PRES.	●●●●
				Non‐verbal behaviours		
				Empathy		
Holmes et al. (2024)	To 1) investigate clinimetric properties of 2 surveys used to evaluate common factors in the patient‐provider relation and 2) present screener options for the assessment of common factors and report their correlation with pain and functional outcomes.	Quantitative observational study.	Patients with acute or chronic MSK pain in spine, upper or lower extremity (*n* = 100).	Task	Common factors screening tools.	●●●
				Bond		
				Treatment credibility		
				Expectations		
Holmes et al. (2024)	To explore participants' perceptions of the influence of common factors on PT outcomes.	Qualitative study with thematic analysis.	Patients with acute or chronic MSK condition affecting different parts of the body (*n* = 7)	An empathetic bond		●●●
				Establishing trust		
				Communication strategies		
				Individualised collaborative care		
				Patient‐centred education		
				Seeing physiotherapy as enjoyable		
				Developed sense of empowerment		
La Touche et al. (2023)	To 1) identify what factors influence the perception of change/recovery through physiotherapy in patients with chronic MSK pain and 2) to compare different types of PT interventions and the perception of change/recovery.	Quantitative observational cross‐sectional study.	Patients with chronic MSK pain (*n* = 150).	Bond	Therapeutic alliance in physiotherapy questionnaire‐patients: CAF‐P.	●●●●
				Collaboration		
Linares‐Fernández et al. (2021)	To present the development and analysis of the factorial structure and psychometric properties of a new self‐administered questionnaire (therapeutic alliance in physiotherapy questionnaire‐patients [CAF‐P]) designed to measure therapeutic alliance in physiotherapy.	Quantitative validation study.	Patients with non‐cancer chronic MSK pain (*n* = 203).	Bond	Therapeutic alliance in physiotherapy questionnaire‐patients: CAF‐P. Working alliance inventory‐short (patient version), Spanish adaptation: WAI‐S‐P.	●●●
				Collaboration		
McCabe, Miciak, Roduta Roberts, Sun, et al. (2022)	To describe the development of a new measure of TR for use in PT, the physiotherapy therapeutic RElationship measure (P‐TREM)	Methodological instrument development study.	Participants involved in the development process in pilot administration with a condition of inflammatory arthritis (*n* = 3) and MSK injury (*n* = 2).	The conditions of engagement: Being present, being receptive, being genuine, and being committed.	The physiotherapy therapeutic RElationship measure: P‐TREM.	●●●●
				Ways of establishing connections: Acknowledging the individual, using the body as a pivot point, and giving‐of‐self.		
				Elements of the bond: caring, trust, respect and nature of the rapport.		
Miciak et al. (2018)	To identify and provide in‐depth descriptions of the necessary conditions of engagement of the TR between physiotherapists and patients.	Qualitative, interpretive description study.	Patients with acute or chronic MSK disorders (*n* = 7). Physiotherapists (*n* = 11).	Conditions fostering engagement between physiotherapist and patient within a TR include present, receptive, genuine, and committed.		●●●●
Miciak et al. (2019)	To identify and provide in‐depth descriptions of the necessary conditions of engagement of the TR between physiotherapists and patients.	Qualitative, interpretive description study.	Patients with acute or chronic MSK disorders (*n* = 7). Physiotherapists (*n* = 11).	Ways of establishing connections in the PT, TR involves acknowledging the individual, giving‐of‐self, and using the body as a pivot point.		●●●●●
Moore et al. (2020)	To investigate participants' experiences of treatment, and barriers and facilitators to exercise and general physical activity behaviour in the longer term.	Qualitative longitudinal study.	Patients with knee pain and/or stiffness in one or both knees and who met criteria for a clinical diagnosis of osteoarthritis (*n* = 30).	Mutual investment		●●
				Personal interactions/affective bond		
				Communication		
Myers et al. (2022)	1) To explore physical therapist behaviours and interactions during the initial PT evaluation and how they related to the patient's perception of TA. 2) to explore the relationship between TA, pain intensity, and function.	A mixed methods study.	Patients with MSK pain condition (*n* = 27). Physiotherapists (*n* = 16).	Information gathering	Working alliance inventory‐short revised: WAI‐SR.	●●
				Patient facilitating		
				Interpersonal		
				Body language		
				Patient support		
				Patient education		
				Language		
				Evidence of agreement		
				Assessing patient understanding		
				Patient centeredness		
				Breakdowns in therapeutic alliance		
Rodríguez Nogueira et al. (2020)	To develop a tool for evaluating person‐centred therapeutic relationships within physiotherapy services, and to examine the content validity of the same.	A mixed methods study.	Patients with non‐specific chronic LBP (*n* = 13).	Personal characteristics of the professional	A tool to evaluate person‐centred therapeutic relationship in physiotherapy services.	●●●
				Communication capacities of the professional		
				Professional aspects		
				Relational aspects		
				Personalised therapy		
				Partnership		
				Environment		
Unsgaard‐Tøndel and Søderstrøm (2021)	To explore how physiotherapists express their experiences of building TA's within a biopsychosocial perspective of LBP.	Qualitative study with hermeneutical design.	Physiotherapists (*n* = 5).	Building trust		●●●
				Facilitating open dialogue		
				Communication		
				Need for advanced clinical reasoning given the complexity of LBP		
				A need for clinical experience as a PT		
				Being an expert on biomechanical issues		
				Aspects of the biopsychosocial models.		
Unsgaard‐Tøndel and Søderstrøm (2021)	To explore patients' expectations before and experiences after PT for LBP.	Qualitative study with hermeneutical design.	Patients with non‐specific chronic LBP (*n* = 13).	Trustworthy and reassuring physiotherapist		●●
				Explaining and empowering		
				Autonomy, taking control		
Zimney et al. (2024)	To (1) quantitatively correlate trust and TA in PT care for patients with chronic low back pain, (2) investigate the relation of trust and TA with outcomes over the course of treatment.	Quantitative cohort study.	Patients with chronic LBP (*n* = 18).	Trust	Working alliance inventory‐short revised: WAI‐SR.	●●

^a^ Dots (●–●●●●●) indicate the number of MMAT criteria rated “Yes” for each study across relevant methodology‐specific questions.

All studies met the quality appraisal's screening criteria, and thus, none were excluded based on the MMAT assessment (Hong, Faberques, et al. 2018). Regarding the methodological quality criteria, ratings varied. Qualitative studies rated well in areas related to the appropriateness of the selected method and the adequacy of data collection methods. Some challenges were identified in the interpretation of the results, mostly in determining the coherence between qualitative data sources, analysis, and interpretation. As a result, some studies did not meet the criteria. All quantitative studies had a valid sampling strategy and appropriate measurements. However, only one quantitative study had an adequately representative sample. The mixed methods studies did not sufficiently outline the rationale for using this study design, but they described the integration of qualitative and quantitative components well.

### 3.1 The Core Components of the TR in MSK Physiotherapy

**FIGURE 2:**
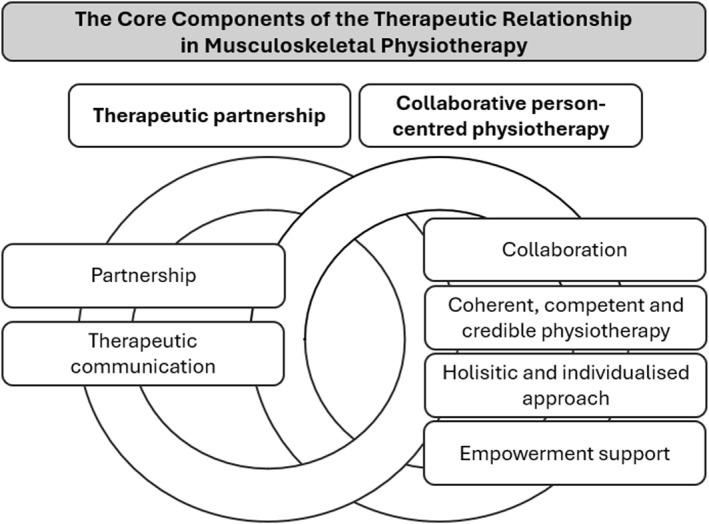
Illustration of the core components and subcomponents of the therapeutic relationship in musculoskeletal physiotherapy.

Two core components of the TR in MSK physiotherapy were identified: the therapeutic partnership and collaborative person‐centred physiotherapy. The therapeutic partnership comprised two subcomponents: partnership and therapeutic communication. Collaborative person‐centred physiotherapy included four subcomponents: collaboration, coherent, competent and credible physiotherapy, a holistic and individualised approach, and empowerment support. Figure 2 illustrates the core components and their subcomponents.

**Therapeutic partnership:** The findings on therapeutic partnership indicate that it is created by a meaningful bond, connection, and mutual engagement between the patient and physiotherapist, established within emotionally supportive, trusting, and safe conditions, and facilitated through various therapeutic communication strategies.

*Partnership* reflects the relational foundation of the TR, characterised by the development of a bond grounded in attentiveness, reciprocal positive feelings, and the patient's sense of being valued (Holmes et al. 2024; La Touche et al. 2023; Linares‐Fernández et al. 2021; Moore et al. 2020). This bond develops through a connection between the patient and physiotherapist, which includes the physiotherapist acknowledging the patient as an individual, equal, and validating their experiences (McCabe, Miciak, Roduta Roberts, Sun, et al. 2022; Miciak et al. 2019; Moore et al. 2020). Engagement within the TR is enhanced when the physiotherapist demonstrates presence, receptiveness, genuineness, and commitment (McCabe, Miciak, Roduta Roberts, Sun, et al. 2022; Miciak et al. 2018). The partnership is further supported by emotional elements such as warmth, respect, and trust (McCabe, Miciak, Roduta Roberts, Sun, et al. 2022; Rodríguez Nogueira et al. 2020). Trust is particularly crucial for forming a partnership (Cosgrove and Hebron 2021; Unsgaard‐Tøndel and Søderstrøm 2021; Zimney et al. 2024); when a patient gains trust and confidence in a physiotherapist, it contributes to the patient's perception of safety within the TR (Calner et al. 2021).

*Therapeutic communication* encompasses verbal, nonverbal, and body communication strategies, techniques, and styles that support the formation of a partnership between the patient and the physiotherapist. Verbal communication involves maintaining an open, ongoing dialogue throughout the physiotherapy process (Calner et al. 2021; Unsgaard‐Tøndel and Søderstrøm 2021). Key strategies include active listening and motivational interviewing (Calner et al. 2021; Fuentes et al. 2014; Rodríguez Nogueira et al. 2020; Unsgaard‐Tøndel et al. 2021; Unsgaard‐Tøndel et al. 2021). The physiotherapist can make the patient central in the process by listening attentively and providing space for the patient's beliefs, thoughts, and questions (Moore et al. 2020; Myers et al. 2022; Unsgaard‐Tøndel and Søderstrøm 2021). Therapeutic communication also involves adjusting the amount and quality of information to suit patient needs, summarising discussions, assessing the patient's understanding, and clarifying if necessary (Myers et al. 2022; Unsgaard‐Tøndel and Søderstrøm 2021). In a therapeutic context, supportive verbal communication is characterised by expressions of empathy (Fuentes et al. 2014; Holmes et al. 2024; Myers et al. 2022). It also involves using language that is patient‐friendly, encouraging, and empowering, together with an appropriate tone and volume to promote clarity and trust (Fuentes et al. 2014; Moore et al. 2020; Myers et al. 2022; Rodríguez Nogueira et al. 2020). When suitable, humour and personal anecdotes further strengthen rapport and support the development of a TR (Myers et al. 2022).

Non‐verbal and body communication involves behaviours that indicate engagement and an embodied presence during therapeutic interaction. Non‐verbal behaviours include maintaining eye contact and adopting specific body positions, such as leaning in or avoiding crossing arms or legs to convey attentiveness and trust (Fuentes et al. 2014; Myers et al. 2022; Rodríguez Nogueira et al. 2020). In physiotherapy, body communication integrates work with the physical body and therapeutic touch as active forms of non‐verbal communication (Fuentes et al. 2014; McCabe, Miciak, Roduta Roberts, Sun, et al. 2022; Miciak et al. 2019). Body communication has been described as ‘using the body as a pivot point’ to explain physical problems and solutions, using therapeutic touch, and facilitating patients' connection to their own bodies (Fuentes et al. 2014; McCabe, Miciak, Roduta Roberts, Sun, et al. 2022; Miciak et al. 2019; Myers et al. 2022).

**Collaborative person‐centred physiotherapy:** *Collaboration*, in the context of a TR, represents the active process through which the patient and physiotherapist work together with equity and reciprocity (La Touche et al. 2023; Linares‐Fernández et al. 2021; Rodríguez Nogueira et al. 2020). Central to collaboration is active patient involvement, supported by the sharing of information and power between the patient and physiotherapist (Beneciuk et al. 2021) and enacted through negotiation and mutual agreement on decisions throughout the physiotherapy process (Miciak et al. 2019; Moore et al. 2020).

Grounded in collaboration, *coherent, competent, and credible physiotherapy* is conceptualised in this review as a logical, person‐centred care process that makes sense to the patient and is delivered with specialised expertise. Coherence emerges when the patient and physiotherapist agree on a treatment plan, mutually set goals, and determine tasks to achieve these goals (Alodaibi et al. 2021; Holmes et al. 2024; Myers et al. 2022). This process is supported by the physiotherapist's professional competence, which combines clinical experience and practical skills. Clinical experience strengthens knowledge and skills, such as advanced clinical reasoning, technical expertise, and the capacity to educate and guide patients, which are needed to manage complex MSK conditions (Rodríguez Nogueira et al. 2020; Unsgaard‐Tøndel and Søderstrøm 2021). Furthermore, the physiotherapist can build credibility by providing clear and understandable information regarding the diagnosis, the condition's effects, and the prognosis. The physiotherapist can also enhance credibility by educating the patient in pain science and clarifying the respective roles of the patient and physiotherapist in managing the condition (Myers et al. 2022). The perceived credibility of the treatment plan and patient expectations of the likely outcomes can influence the effectiveness of the treatment, either enhancing or undermining it (Holmes et al. 2024).

*Holistic and individualised approaches* to developing the TR include the application of the biopsychosocial model (BPSM) to patient‐specific care strategies. The core of person‐centred physiotherapy is a holistic understanding of how a condition affects an individual and their life biopsychosocially (Myers et al. 2022; Unsgaard‐Tøndel and Søderstrøm 2021). Individualised physiotherapy begins by giving the patient time to tell their own story, and includes personalised education, exercises, and care (Calner et al. 2021; Holmes et al. 2024). To facilitate a holistic and individualised approach, the therapeutic environment should support the physiotherapist's professional autonomy, and promote multiprofessionalism (Rodríguez Nogueira et al. 2020).

*Empowerment* *support* refers to strategies that aim to empower the patient, thus strengthening their confidence and ability to manage their condition actively. In physiotherapy practice, this involves facilitating treatment adherence through meaningful self‐management approaches, such as educating the patient about the body, providing individualised information, advice and tools, adjusting their physical activity; and providing strategies for building tolerance and managing pain (Holmes et al. 2024; Unsgaard‐Tøndel and Søderstrøm 2021). It also involves offering patients reassurance, fostering motivation, and providing encouragement, thereby enhancing their engagement in the therapeutic process (Holmes et al. 2024; Rodríguez Nogueira et al. 2020; Unsgaard‐Tøndel and Søderstrøm 2021). Supporting empowerment may foster patients' autonomy and enable them to control their everyday lives better (Unsgaard‐Tøndel and Søderstrøm 2021).

### 3.2 Patient‐Reported Instruments for Assessing the TR in MSK Physiotherapy

We identified six different patient‐reported instruments, in use or in development for assessing the TR in MSK physiotherapy. The instruments and their characteristics are summarised in Table 4.

**TABLE 4:** Patient‐reported instruments for assessing the TR in MSK physiotherapy.

Patient‐reported instrument	Focus	Subscales and items	References
Working alliance inventory‐short revised: WAI‐SR	Working alliance	3 subscales: Bond, goals, tasks.	Alodaibi et al. (2021); Beneciuk et al. (2021); Holmes et al. (2024); Linares‐Fernández et al. (2021); Myers et al. (2022); Zimney et al. (2024)
		12 items	
Therapeutic alliance in physiotherapy questionnaire‐patients: CAF‐P	Therapeutic alliance	2 subscales: Bond and collaboration.	La Touche et al. (2023); Linares‐Fernández et al. (2021)
		14 items	
Pain rehabilitation expectations scale: PRES (working alliance subscale)	Working alliance	Not reported	Fuentes et al. (2014)
Common factors screening tools	Common factors	4 subscales: Bond, task, credibility, and expectations.	Holmes et al. (2024)
		11 items.	
		Reduced version: 3 items.	
The physiotherapy therapeutic RElationship measure: P‐TREM (in development)	Therapeutic relationship	3 subscales: Elements of bond, ways of establishing connections, and conditions of engagement.	McCabe, Miciak, Roduta Roberts, Sun, et al. (2022)
		49 items.	
A tool to evaluate the person‐centred therapeutic relationship in physiotherapy services (in development)	Therapeutic relationship	7 subscales: Professional's personal characteristics, Professional's communication capacities, professional aspects, relational aspects, personalised therapy, partnership, and environment.	Rodríguez Nogueira et al. (2020)
		31 items	

The Working Alliance Inventory‐Short Revised (WAI‐SR) was the most frequently used instrument across the studies. It was originally developed as a psychotherapy tool and was later adapted for physiotherapy contexts (Alodaibi et al. 2021; Beneciuk et al. 2021; Holmes et al. 2024; Linares‐Fernández et al. 2021; Myers et al. 2022; Zimney et al. 2024). The Therapeutic Alliance in Physiotherapy Questionnaire—Patients (CAF‐P) was specifically designed for physiotherapy context and has been validated in chronic MSK pain populations (La Touche et al. 2023; Linares‐Fernández et al. 2021). The Working Alliance of the Pain Rehabilitation Expectations Scale (PRES) subscale ‘working alliance’ was used in one study to capture WA aspects (Fuentes et al. 2014). The Common Factors Screening Tool integrates WA aspects along with measurements of the patient's belief in the logic of a treatment (credibility) and expectation of an improvement in their condition (expectancy); it is available in short and long versions (Holmes et al. 2024.). The Physiotherapy Therapeutic RElationship Measure (P‐TREM) and ‘A tool to evaluate the person‐centred therapeutic relationship in physiotherapy services’ were developed to incorporate physiotherapy‐specific TR elements. However, both instruments are still in development (McCabe, Miciak, Roduta Roberts, Sun, et al. 2022; Rodríguez Nogueira et al. 2020).

## 4 Discussion

This systematic integrative review synthesised the evidence on the core components of the TR and the instruments used to assess it in the context of MSK physiotherapy. Two core components were identified: therapeutic partnership and collaborative person‐centred physiotherapy. These components reflect both relational aspects and those integrated within physiotherapy practice. Six patient‐reported instruments were also identified. These findings corroborate previous work identifying similar components of the TR, while offering new insights into its dynamic nature (Kinney et al. 2020; O’Keeffe et al. 2016). Our results highlight the importance of communication in building partnerships and the central role of person‐centred care in promoting collaborative, holistic, and empowering physiotherapy practice. Thus, they contribute to a physiotherapy‐specific understanding of TR and offer new insights into TR assessment in MSK physiotherapy.

The findings on *therapeutic partnership* indicate that relational elements, such as a meaningful bond and mutual engagement between the patient and physiotherapist, develop most effectively in emotionally supportive, trusting, and safe conditions, enhanced by therapeutic communication. These findings contribute to the evidence that a physiotherapist's communication style is central to fostering the TR in MSK physiotherapy, aligning with studies in other physiotherapy and rehabilitation contexts (Babatunde et al. 2017; Søndenå et al. 2020). In a review by O’Keeffe et al. (2016), interpersonal and communication skills were identified as the most influential factors shaping therapeutic interactions in MSK physiotherapy. Beyond verbal communication, such as active listening and motivational interviewing, the present review highlights the importance of body communication as a physiotherapy‐specific aspect within MSK care.

Body communication involves the intentional use of therapeutic touch, movement, and an embodied presence to support the relational processes that shape the TR in MSK physiotherapy (Ahlsen and Nilsen 2022; Good et al. 2024). Therapeutic touch should therefore be recognised as more than a technical skill or mechanical procedure; it is a central resource facilitating the patient‐therapist interaction (Ahlsen and Nilsen 2022). For example, according to Good et al. (2024), therapeutic touch can influence patients' perceptions of the physiotherapist's engagement and competence, strengthening trust and confidence. In line with these findings, a study in the present review reported that the absence of human touch was associated with a weaker TR (Myers et al. 2022). Similar findings emerge in remotely delivered MSK physiotherapy contexts, where a TR can be established, but it is typically less developed than in in‐person contexts, often due to limited opportunities for hands‐on interaction (Fritz et al. 2022). These findings emphasise that in fostering TR in MSK physiotherapy, the role of therapeutic touch and embodied communication is valuable.

The findings on *collaborative person‐centred physiotherapy* suggest that the TR is integrated within physiotherapy practice and shares multiple aspects with person‐centred care. These findings corroborate earlier literature, which indicated that the TR is important for unifying person‐centred practices in clinical encounters, highlighting the reciprocal nature of the TR and person‐centred care (Hamovitch et al. 2018; Morera‐Balaguer et al. 2021). For example, practices such as fostering shared decision‐making, ensuring congruence in physiotherapy process delivered with specialised expertise, acknowledging biopsychosocial and individual needs, and supporting patient empowerment simultaneously reflect person‐centred care and strengthen the TR. Recognising this connection offers physiotherapy‐specific insights on how the TR can be enhanced by embedding person‐centred care practices in clinical encounters. Taken together, this suggests that the TR and person‐centred care are mutually reinforcing concepts, through which the TR is built, maintained, and strengthened.

The findings highlight that adopting a holistic BPSM supports the development of a stronger TR in MSK physiotherapy (Myers et al. 2022; Unsgaard‐Tøndel and Søderstrøm 2021). While the importance of individualised care has been identified in the previous reviews by O'Keeffe et al. (2016) and Kinney et al. (2020), a holistic approach is a less explored element of the TR in MSK physiotherapy. Despite the recognised value of implementing the BPSM as a standard approach in MSK physiotherapy, many physiotherapists report challenges in addressing psychosocial issues in clinical reasoning and practice, and express concerns about professional boundaries (Holopainen et al. 2020; van Dijk et al. 2023). This hesitation on the part of health care professionals underscores the need for ongoing research and education to effectively integrate the BPSM into physiotherapy practice, ensuring holistic and individualised care for patients with MSK conditions.

Empowerment support in a TR consists of employing strategies that enhance patients' confidence and capacity for active management of their MSK conditions. In the included studies, patients described many practical strategies to support their self‐management, such as individualised education, exercise, and activity adjustment. On the other hand, they also emphasised the importance of motivational and psychosocial support. For example, a physiotherapist with a trustworthy and reassuring manner reduced fear in patients and strengthened their belief that they could cope with their condition and participate in everyday activities. Patients also reported that feeling validated, having their progress acknowledged, and perceiving their assigned exercises as meaningful contributed to their sense of being seen. This, in turn, enhanced their engagement in exercise and supported sustained adherence (Holmes et al. 2024; Unsgaard‐Tøndel and Søderstrøm 2021). However, evidence from a recent qualitative study by Cioeta et al. (2026) shows that in MSK practice, physiotherapists most commonly operationalise self‐management support by providing information, offering education, and suggesting therapeutic exercise, rather than acknowledging the need for motivational and psychosocial support. These insights suggest that a combination of practical and psychosocial empowerment strategies strengthens the TR in MSK physiotherapy by supporting patients' agency and confidence in managing their conditions.

The instruments identified for assessing the TR in MSK physiotherapy reflect the construct's multidimensional nature. All instruments included ‘bond’ or ‘partnership’, as elements, emphasising the importance of the TR's relational aspects. Most of the instruments included components related to the treatment process, such as ‘goals’, ‘tasks’, ‘collaboration’, and ‘personalised therapy’. In contrast, only one instrument included elements related to communication or physiotherapy‐specific components (Rodríguez Nogueira et al. 2020). None of the instruments included all of the TR subcomponents identified in this review; elements related to a holistic approach and patient empowerment were notably absent. These findings reflect the multidimensionality of the TR and demonstrate that its conceptualisation in physiotherapy is still fragmented and not yet fully established (Cole et al. 2025).

We observed a growing interest in developing new instruments to assess TR in MSK physiotherapy. Five of the identified instruments were developed for the physiotherapy context, four of them in the period 2020–2024. These instruments were not identified in previous reviews exploring the instruments used to assess the TR in physiotherapy (Babatunde et al. 2017; Gutiérrez‐Sánchez et al. 2021). This interest likely reflects the recognition that instruments derived from psychotherapy may not fully capture the characteristics of a physiotherapy‐specific TR. It also aligns with the growing acknowledgement that the TR and person‐centred care are interrelated mechanisms that influence clinical outcomes. This trend, however, is not unique to physiotherapy. A recent study of the nursing field found that in mental health settings, the TR is assessed using instruments originally developed for psychotherapy (Robinson et al. 2025); the authors highlight the importance of developing context‐specific instruments, as TR elements may vary across healthcare settings. Overall, these insights suggest that instruments capable of capturing the unique, context‐specific elements of the TR are needed. The development of such instruments would support healthcare professionals and researchers in assessing, monitoring, and optimising the TR across all settings, including MSK physiotherapy.

### 4.1 Strengths and Limitations

The strengths of this review include its adherence to the principles of the integrative review methodology. This approach enabled the inclusion and synthesis of diverse study designs. Additionally, the review's search strategy was comprehensive, and developed in consultation with a senior information specialist. This review's study population was extensive, as the included studies covered a wide range of patients with various acute and chronic MSK conditions, as well as the physiotherapists treating such patients. This broad approach enabled the identification of commonalities in the core components of the TR. In addition, the inductive qualitative content analysis supported a systematic organisation of the extracted data and contributed to the identification and structured description of the core components of the TR and the instruments used to assess it in MSK physiotherapy.

This review also has limitations. Due to the exclusion criteria (language and date) and the inclusion of only primary empirical studies, some relevant data may have been omitted. The included studies also presented their results in a heterogeneous manner; their descriptions of the core components of TR ranged from single words or sentences in quantitative studies to very comprehensive and detailed descriptions in qualitative and mixed methods studies. This variability may have contributed to some overlap between subcomponents, and some subcomponents (e.g., therapeutic communication) may have received more detailed descriptions than others (e.g., collaboration). Furthermore, the review's broad context may have led to a loss of nuance in distinguishing between differing aspects of the TR in specific, acute, or chronic MSK conditions. This last point is important to acknowledge, as the TR may have different emphases in different contexts.

## 5 Conclusions

This systematic integrative review offers a comprehensive overview of the TR in MSK physiotherapy, identifying its core components and the instruments used to assess it. The synthesis demonstrates that the TR is a multidimensional and dynamic construct encompassing therapeutic partnership and collaborative person‐centred physiotherapy. The findings suggest that physiotherapists can actively develop and maintain the TR with their patients by utilising multimodal communication, implementing person‐centred care strategies, such as shared decision‐making, holistic and individualised care, and supporting patients' autonomy and empowerment. Additionally, we found that although several patient‐reported TR assessment instruments exist, these do not sufficiently represent important physiotherapy‐specific components. Given that assessing the TR is essential for the development and optimisation of therapeutic conditions and practice in physiotherapy, further development of context‐specific and patient‐informed instruments is needed.

Further research should prioritise incorporating patients' perspectives on meaningful TR components and on the process through which the TR develops. By doing so, it would strengthen conceptual clarity and refine both the operationalisation and assessment of the TR in MSK physiotherapy.

## Author Contributions

**Sini Puustinen:** conceptualization, methodology, data curation, formal analysis, visualization, writing – original draft, writing – review and editing. **Tarja Kvist:** formal analysis, writing – review and editing, supervision. **Minna Stolt:** formal analysis, writing – review and editing, supervision.

## Funding

The authors have nothing to report.

## Conflicts of Interest

The authors declare no conflicts of interest.

## Data Availability

The authors have nothing to report.
